# Herpes reactivation after the injection of hyaluronic acid dermal filler: A case report and review of literature

**DOI:** 10.1097/MD.0000000000020394

**Published:** 2020-06-12

**Authors:** Chenyu Wang, Tianyu Sun, Nanze Yu, Xiaojun Wang

**Affiliations:** a Department of Plastic surgery, Peking Union Medical College Hospital, Peking Union Medical College and Chinese Academy of Medical Sciences, Beijing, 100730, China; b Nuffield Division of Clinical Laboratory Sciences, Radcliffe Department of Medicine, University of Oxford, Oxford, OX3 9DU, UK.

**Keywords:** cosmetic injection, herpes reactivation, hyaluronic acid

## Abstract

**Introduction:**

Hyaluronic acid injections is relatively safe with little risk of complications. Although herpes reactivation after the injection of hyaluronic acid is rare, it produces quite a huge pressure and panic on patients. Quite a lot cosmetic practitioners have no awareness of preventing, diagnosing, and giving correct treatment in time due to lack of experience.

**Patient concerns:**

A 24-year-old woman presented with erythema, crusted papules, pain and swelling on the nose after receiving the injection of hyaluronic acid. A swab of the discharge fluid was obtained for bacterial and viral culture, showing positive for herpes simplex virus.

**Diagnosis:**

The patient was diagnosed as herpes reactivation after the injection of hyaluronic acid.

**Interventions:**

The patient underwent antiviral therapy with acyclovir 400 mg, 3 times daily for seven days.

**Outcomes:**

After a week of antiviral treatment, the clinical signs improved.

**Conclusion:**

Herpes reactivation after the injection of hyaluronic acid is quite rare but needed sufficient attention of cosmetic practitioners to make the proper diagnosis, prevention and treatment.

## 1 Introduction

Injection of dermal fillers is the most frequent nonsurgical cosmetic procedure performed in the world. Injectable facial dermal fillers are an option in the treatment of age-related soft tissue loss, depressed scars, facial sculpting and contouring, augmentation of specific anatomical sites such as the lips and nose, and atrophy or asymmetry induced by disease.^[1,2]^ Dermal fillers are increasingly used for aesthetic purposes in clinical practice, especially hyaluronic acid. Hyaluronic acid is a non-sulfated glycosaminoglycan polysaccharide composed of repeating disaccharide units of glucuronic acid N-acetylglucosamine. Hyaluronic acid injections are relatively safe with short recovery time and little risk of complications when given by experts.^[3]^ There are common complications following filler injections, such as bruising, itching, and unsatisfactory shape. Relatively rare complications include allergic reactions, vascular embolism, and herpes infection.^[4]^ Although herpes reactivation after the injection of hyaluronic acid is rare, it produces quite a considerable pressure and panic on patients. Quite a lot of cosmetic practitioners have no awareness of preventing, diagnosing, and giving correct treatment in time due to lack of experience. With this, we shared a case and literature review of herpes reactivation after the injection of hyaluronic acid aimed to explore appropriate assessment to diagnose, prevent, and treat such a rare complication.

## 2 Case report

**Figure 1:**
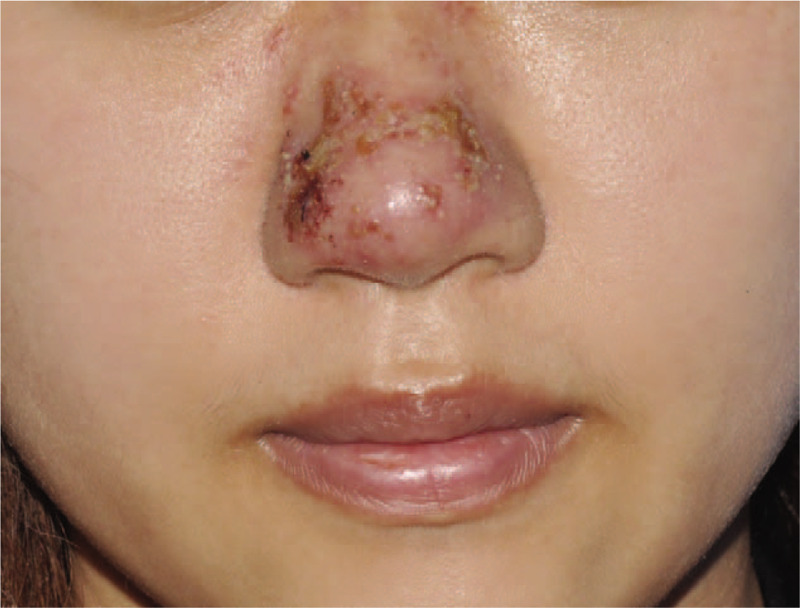
A 24-yr-old woman presented with erythema and crusted papules on the nose after injection of hyaluronic acid filler.

A 24-year-old woman presented to our clinic with the chief complaint of erythema, crusted papules, pain and swelling on the nose for four days. She received an injection of hyaluronic acid into her nose (Restylane, PerlaneTM, Sweden,1 mL), for aesthetic purposes. A vesicle appeared on the nose the other day after the injection, and angioedema-like swelling, erythema, and crusted papules appeared later. (Fig. 1) She asked the consultant in our clinic. A swab of the discharge fluid was obtained for bacterial and viral culture. The result proved positive for herpes simplex virus. She reported no herpes outbreaks history. Therefore, no antiviral prophylaxis was not performed before filler injection. The patient underwent antiviral therapy with acyclovir 400 mg, three times daily for seven days. After a week of antiviral treatment, the clinical signs improved. The patient has signed the informed consent.

## 3 Discussion

### 3.1 Literature review

To further investigate the herpes reactivation after the injection of hyaluronic acid, a PubMed, Embase, and Medline database search was performed. Articles selected were published from 1990 to August 2019 with the keywords of “herpes, herpes simplex virus (HSV), herpes zoster, hyaluronic acid.” Four articles with 4 cases reported were enrolled (Table 1).^[5–8]^

**Table 1:**
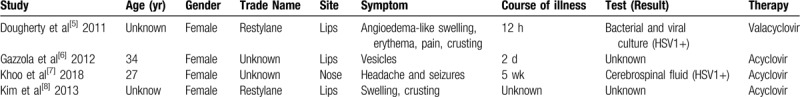
Information of cases with herpes reactivation after hyaluronic acid injection.

### 3.2 Etiology and symptoms

Dermal fillers injection for cosmetic procedures is increasingly performed, whose complications have been described in the literature, including bruising, itching, infections, and tissue necrosis. Relatively rare complications include allergic reactions, vascular embolism, and herpes infection. The risk of herpes reactivation after dermal filler injection is rare, with an incidence of herpes simplex virus 1 (HSV-1) reactivation estimated to be less than 1.45% cases and herpes zoster virus even rarer.^[9]^ There are 8 separate species in the herpes family of viruses that infects humans: HSV-1, HSV-2, varicella-zoster virus, Epstein-Barr virus, cytomegalovirus, human herpesvirus 6, human herpesvirus 7, and human herpesvirus 8. These viruses all have double-stranded linear deoxyribo nucleic acid enclosed within an icosahedral capsid, protein tegument, and lipid envelope.^[5,6]^ These viruses have to replicate deoxyribo nucleic acid and transcript with a subsequent synthesis of the gene products within the host cell nucleus. Therefore, the characteristic cytologic inclusions are located within the nucleus. Active virus replication and host immune response mostly lead to acute clinical presentation. Acute symptoms include angioedema-like swelling, erythema, local pain, and crusting. A decrease in viral load accompanies the improvement in symptoms. However, the resolution of symptoms does not herald the clearance of the virus. Sometimes, the infection persists within the host. Once the conditions are satisfied, the infection may break out again.^[7,8,10]^ Some reports demonstrated that hyaluronic acid itself has the advantage of being a protective agent, preventing viral replication.^[11]^ The antiviral efficacy can be correlated with the degree of sulfation and becomes stronger with increasing the degree of sulfation values.^[12]^ We summarize that there may be 3 mechanisms to provoke virus reactivations and lead to herpes outbreaks after injection of hyaluronic acid:

(1) local trauma, such as the direct damage to the axon by the needle and the tissue manipulation after filler injection;
(2) inflammatory reaction after filler injection;
(3) systemic stress or immunosuppression.

Virus reactivation usually appears in the area where the filler has been injected, mostly, perioral area and nasolabial folds. Virus activation is commonly observed 24 to 48 hours after filler injection.^[5–7]^ However, in some cases, virus reactivation can extend and affect neighboring areas. It is believed that this pattern of reactivation is determined by the infective agent, mainly HSV-1 and HZV. Khoo CS reported a case of HSV-1 Encephalitis after nasal dermal filler injection.^[13]^ Due to its affinity for nerve cells, HSV viruses lie dormant in the dorsal root ganglia after the initial infection and may become reactivated at a later stage, causing meningitis or encephalitis.

### 3.3 Diagnosis

Symptoms of herpes reactivation after filler injection mostly are angioedema-like swelling, erythema, local pain and crusting, which are usually observed 24 to 48 hours after filler injection. Mostly, it's needed to be distinguished from allergic reactions. Acute allergic reactions of hyaluronic acid usually occur within 48 hours of injection, typically manifested as local inflammatory or non-inflammatory swelling at the injection site, local skin changes, and etc. Histopathologic examination of hematoxylin-eosin–stained slides usually will not be sufficient for diagnosis. Viral culture, which is still regarded as the gold standard, is time-consuming and has a lower sensitivity compared with other methods. Tzanck smears for HSV and Varicella-zoster virus are easily and rapidly prepared in the clinical setting and, when positive, allow for quick, inexpensive diagnosis.^[14]^ We summarized the conditions of diagnosis for herpes reactivation after the injection of hyaluronic acid:

(1) The patient has a history of facial injection of hyaluronic acid recently (within 48 hours).
(2) The patient may report no history of herpes.
(3) The patient has skin lesions of herpes attacks or neurological symptoms of encephalitis.
(4) The experimental examination shows positive evidence.

### 3.4 Therapy

Therapy for symptomatic HSV relies on antiviral drugs: acyclovir, valacyclovir hydrochloride, and famciclovir. The current therapeutic approach can be divided into three groups: intermittent episodic therapy (IET), chronic suppressive therapy, and intermittent suppressive therapy. IET is administered for acute episodes of chronic disease, chronic suppressive therapy is applied for frequent recurrences with severe functional impairment and disfigurement, and intermittent suppressive therapy is used to prevent oral and genital herpes for short periods. In cases of HSV outbreak, IET should be administered, with 400 mg acyclovir three times per day for ten days or 1 g valacyclovir hydrochloride twice per day for 7 days.^[15]^

### 3.5 Prevention

The cosmetic practitioners should gain an accurate medical interview, including those symptoms that could underline a previous herpes outbreak (vesicles, erythema, desquamation), localization, and past procedures with filler injection.^[16]^ A proper clinical examination and a strict follow-up after the injection are essential to prevent and treat any herpes reactivation effectively. Oral prophylaxis should be recommended to the patients who had herpes history or in poor immune condition, with 400 mg acyclovir three times per day for ten days or 1 g valacyclovir twice per day for 7 days.^[17]^

## 4 Conclusion

Herpes reactivation after the injection of hyaluronic acid is quite rare but needed sufficient attention of cosmetic practitioners to make the proper diagnosis, prevention, and treatment.

## Author contributions

**Conceptualization:** Xiaojun Wang.

**Data curation:** Chenyu Wang.

**Formal analysis:** Chenyu Wang, Nanze Yu.

**Methodology:** Tianyu Sun.

**Writing – original draft:** Chenyu Wang, Tianyu Sun.

**Writing – review & editing:** Xiaojun Wang.
